# Detecting N^6^-methyladenosine sites from RNA transcriptomes using ensemble Support Vector Machines

**DOI:** 10.1038/srep40242

**Published:** 2017-01-12

**Authors:** Wei Chen, Pengwei Xing, Quan Zou

**Affiliations:** 1 School of Science, Center for Genomics and Computational Biology, North China University of Science and Technology, Tangshan 063009, China; 2 School of Computer Science and Technology, Tianjin University, Tianjin 300354, China; 3 State Key Laboratory of Medicinal Chemical Biology, NanKai University, Tianjin 300074, China

## Abstract

As one of the most abundant RNA post-transcriptional modifications, N^6^-methyladenosine (m^6^A) involves in a broad spectrum of biological and physiological processes ranging from mRNA splicing and stability to cell differentiation and reprogramming. However, experimental identification of m^6^A sites is expensive and laborious. Therefore, it is urgent to develop computational methods for reliable prediction of m^6^A sites from primary RNA sequences. In the current study, a new method called **RAM-ESVM** was developed for detecting m^6^A sites from *Saccharomyces cerevisiae* transcriptome, which employed ensemble support vector machine classifiers and novel sequence features. The jackknife test results show that RAM-ESVM outperforms single support vector machine classifiers and other existing methods, indicating that it would be a useful computational tool for detecting m^6^A sites in *S. cerevisiae*. Furthermore, a web server named RAM-ESVM was constructed and could be freely accessible at http://server.malab.cn/RAM-ESVM/.

Among the ~150 kinds of RNA modifications identified in cellular RNA^1^, N^6^-methyladenosine (m^6^A) is the most abundant one and is catalyzed by N^6^-adenosyl methyltransferases including METTL3, METTL14 and WTAP^2^. Since it was discovered in 1970s, m^6^A has been found from bacteria to *Homo sapiens*^1^. Recent studies have suggested that m^6^A joined a series of molecular processes such as protein translation and localization^3^, and even contributed to obesity^4^, brain development abnormalities and other diseases^5^. As indicated in a recent study^6^, m^6^A is non-randomly distributed in the genome. Thus, the knowledge about the positions of m^6^A site is important for understanding its biological functions.

Attribute to the high-throughput experimental techniques, the genome-wide distribution of m^6^A are now available for several species, such as *Saccharomyces cerevisiae*^6^, *Arabidopsis thaliana*^7^, *Mus musculus*^8^ and *Homo sapiens*^8^. Recently, Jaffrey and his colleagues provided the single-nucleotide resolution map of the m^6^A sites across human transcriptome by using the miCLIP technique^9^. However, the resolution of m^6^A sites for other species is not fully satisfactory, i.e. they couldn’t pick out the modified adenosine residue sites. Moreover, wet experiments are laborious in performing genome-wide m^6^A sites detections. So it is essential and necessary to employ novel computational approaches for detecting m^6^A sites. *In silico* approaches would also do the detection in genome-wide scale and could help to save the wet experiments cost.

The high-resolution experimental data provided unprecedented opportunities and made it feasible to develop computational methods for accurately predicting m^6^A sites. Depending on these data, various computational methods have been proposed to identify m^6^A sites. By encoding RNA sequence using nucleotide chemical property and pseudo nucleotide composition, Chen *et al*. have proposed two yeast-specific m^6^A site prediction webservers^10^,^11^. Inspired by Chen *et al*.’s works^10^,^11^, Zhou and his co-workers also proposed a mammalian m^6^A site predictor named SRAMP^12^. Subsequently, a webserver called MethyRNA was proposed to identify m^6^A sites in *H. sapiens* and *M. musculus*^13^. Although the performances of existing methods are satisfactory for identifying m^6^A site in mammalian transcriptomes^13^, they fails to accurately identify m^6^A site in yeast^12^. This may be due to the fact that the information around the yeast m^6^A site has not been fully characterized^12^. More recently, Zhang *et al*. improved the performance of identifying m^6^A site in yeast by introducing the heuristic nucleotide physical-chemical property selection algorithm^14^. However, the performance for identifying m^6^A site in yeast transcriptome is still not satisfactory and should be improved further.

Keeping this in mind, in the present study, we proposed an ensemble classifier, called **RAM**-**ESVM**, for detecting m^6^A sites in *S. cerevisiae*. **RAM**-**ESVM** combined three basic classifiers, namely SVM-PseKNC, SVM-motif and GkmSVM^15^, which were constructed by using PseKNC^16^,^17^, motif features, and optimized K-mer as the features, respectively. The predictive results obtained on the benchmark dataset demonstrate that **RAM**-**ESVM** can obviously improve the predictive performance by combining various features and also outperforms the existing methods.

## Result and Discussion

### Comparison of different feature extraction strategies

In order to demonstrate the effectiveness of PseDNC and motif features for m^6^A sites prediction, we compared the performance of PseDNC and motif features with other RNA sequence features. Xue *et al*.^18^ have proposed 32-D (dimensional) triplet features for microRNA precursor identification. The 32-D features include RNA secondary structure information and are proved to represent RNA sequence well. More recently, Wei *et al*.^19^ developed the RNA sequence numeric fingerprints to 98-D, which was proved to be more robust for human microRNA detection. The 98-D features not only include Xue’s 32-D features and but also include free energy features. Therefore, we employed the SVM to perform the comparisons between the models based on our PseDNC and motif features with that based on the 32D and 98D features. Their jackknife test results are showed in Table 1. We can see that the model based on motif features yielded the best predictive accuracy. The performance of the model based on PseDNC is comparable with that based on the 98D features. However, the PseDNC could include local and global sequence order information with a lower dimension. Therefore, PseDNC and motif features were used to encode the samples in the current work.

### Comparison of SVM and other classifiers

To demonstrate the superiority of using SVM for identifying m^6^A sites, we compared its performance with that of other methods, such as Random Forest (RF), K-Nearest Neighbor (KNN), J48 and Naïve Bayes. Inspired by a previous study^11^, the other classifiers were implemented in WEKA^20^ with their default parameters. Table 2 showed the jackknife test comparison of m^6^A sites prediction accuracy in the benchmark dataset. We noticed that the predictive accuracy (Acc) and mathew’s correlation coefficient (MCC) of SVM are superior to those of other methods by using the PseDNC and motif features, respectively. Therefore, the SVM was used to build computational models in the followings.

### Comparison of ensemble SVM with single SVM

Several works suggested that ensemble classifier would improve the performance^21^,^22^,^23^. Here, we employed PseDNC features together with SVM, motif features together with SVM, and GkSVM as three basic classifiers. They vote for the final results. Table 3 shows the performance comparison in detail. We found that the ensemble SVM worked better and improved nearly 10 percent from the basic classifiers for identifying m^6^A sites. Therefore, a m^6^A site predictor, called **RAM**-**ESVM**, was developed based on the ensemble SVM, where “R” stands for RNA, “A” stands for N^6^-adenosine, “M” stands for methylation, “E” stands for Ensemble, “SVM” stands for Support Vector Machine.

### Comparison of RAM-ESVM with existing method

To the best of our knowledge, M6A-HPCS^14^ is the best predictor for identifying m^6^A sites in yeast. In order to further verify the power of **RAM-ESVM** on the m^6^A prediction task, we compared its performance with that of M6A-HPCS by using the same benchmark dataset as that used in the current work. From Table 4 we could conclude that the rates for *Sn, Sp, Acc* and *MCC* of **RAM**-**ESVM** are all higher than that of M6A-HPCS, indicating that **RAM-ESVM** is quite promising for identifying m^6^A sites.

### Web server description

**Figure 1:**
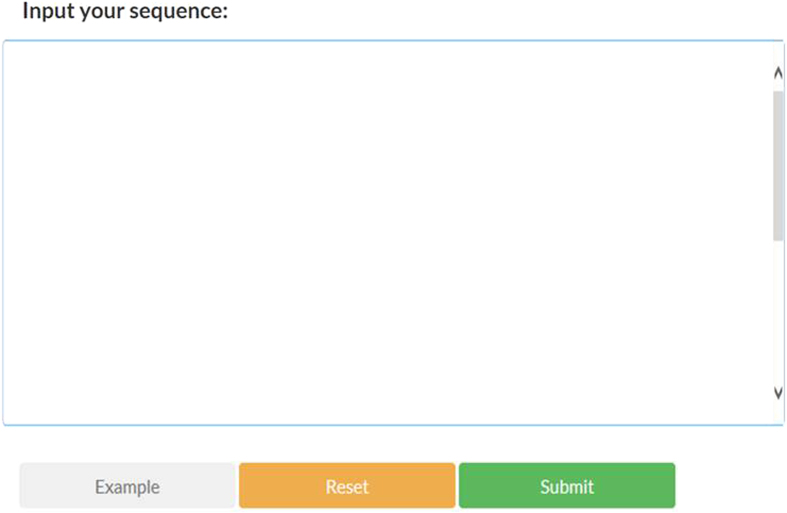
A semi-screenshot for the top-page of the RAM-ESVM web-server at http://server.malab.cn/RAM-ESVM/.

In order for the conveniences of scientific community, a freely accessible online web-server of **RAM-ESVM** was established, which could benefit for the biological researchers. Its top-page is shown in Fig. 1.

The users can either paste or type their query RNA sequences for submission, which should be with FASTA format. By clicking the “Submit” button, the predictive results will be shown in a new page and the detected m^6^A sites will be indicated in red. For the user’s convenience, the results can also be saved in tab-delimited text format by clicking the “Fasta Format Result” button.

## Conclusions

m^6^A plays important roles in many biological processes. With the rapid increase in amount of transcriptome data, there is a growing need for developing efficient and reliable computational methods to accurately identify m^6^A sites. In the present work, a new predictor, called **RAM-ESVM**, was developed to identify m^6^A sites, which is based on an ensemble of support vector machine classifiers. Although SVM was chosen as the classifier, the features are heterogeneous. The first one employed PseKNC features, which are always used in RNA/DNA classification. The second one is motif features, which is proposed first time in this work. The third one is a string classifier. It avoided feature extraction for RNA sequences. They modified the kernel computation and deal with the strings as vectors. The jackknife test results demonstrate that **RAM-ESVM** is very promising and outperforms M6A-HPCS which is the best of the existing web servers for m^6^A sites detection in *S. cerevisiae*.

The better performance of **RAM-ESVM** could be attributed to the following reasons. In **RAM-ESVM**, not only the sequence local and global sequence information was included by encoding RNA sequences using PseKNC, but also the sequence motifs were considered. Since the m^6^A is catalyzed by N^6^-adenosyl methyltransferases, the sequence motifs determined by MEME and DMINDA may be the binding targets of the N^6^-adenosyl methyltransferases.

In order to benefit for the vast majority of biology scientists, a user-friendly web server named **RAM-ESVM** has been established at http://server.malab.cn/RAM-ESVM/, by which users can easily obtain their desired results. It is anticipated that **RAM**-**ESVM** will become an essential software tool for identifying m^6^A in yeast.

## Materials and Methods

### Dataset

The benchmark dataset in this paper was obtained from our previous work^10^, which contains 1,307 positive sequences (containing m^6^A sites) and 1,307 negative sequences (non m^6^A sites). The 1,307 positive samples were experimentally identified m^6^A sites. In order to balance the training set, the 1,307 negative samples were randomly picked out from the 33,280 non-m^6^A sites. All the positive and negative samples are 51-nt with the sequence similarity less than 85%.

### Sequence encoding schemes

The merits of multi view learning have been demonstrated in several weak classification problems. Therefore, in order to include the genomic information as much as possible, two kinds of features were used to build SVM classifiers. The first kind of feature is pseudo nucleotide composition. The other one is the gapped sequence motif features. These two kinds of features were extracted with different views. Their definitions are as following.

#### Pseudo nucleotide composition

In order to formulate the sequences using a mathematical expression that can truly reflect their intrinsic correlation with the target to be predicted, the pseudo nucleotide composition (PseKNC) has been proposed^16^,^17^. By using PseKNC, both the local and global sequence order information could be included^24^. Accordingly, the pseudo dinucleotide composition (PseDNC) was used to represent the RNA sequences in the benchmark dataset and can be defined as,


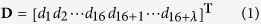


where


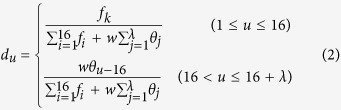


where *f*_*k*_ 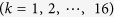 is the normalized occurrence frequency of the non-overlapping dinucleotides in RNA sequence. *λ* is the number of the total counted ranks (or tiers) of the correlations along a RNA sequence, and 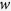 is the weight factor; while the correlation factor *θ*_*j*_ represents the *j*-tier structural correlation factor between all the *j-*th most contiguous dinucleotide D_*i*_ = R_*i*_R_*i*+1_ and is defined as,


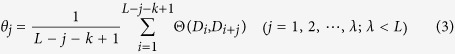


The correlation function Θ(D_*i*_, D_*j*_) is given by


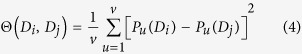


where *v* is the number of RNA physicochemical properties.

Since the formation of RNA secondary structure decreases the m^6^A methylation^6^, the following three physicochemical properties, namely enthalpy^25^, entropy^25^ and free energy^26^ that can quantify the RNA secondary structures, are used to calculate the global or long-range sequence-order effects. Hence, *v* equals to 3 and indicates three kinds of physicochemical properties were considered in the current study. The concrete values of the three physicochemical properties are listed in Table 5. Note that before substituting them into Eq. 4, all the original values were subjected to a standard conversion, as described by the following equation


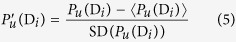


where the symbol < > means taking the average of the quantity therein over the 16 different dinucleotides, and SD means the corresponding standard deviation.

In order to reduce the computational time, the 10-fold cross-validation approach was used to optimize the two parameters. We found that the optimal values for 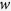 and 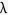 are 0.9 and 6, respectively.

#### Motif features

Motifs are considered as sequence signal for several genomic elements, such as gene Transcription Starting Sites (TSS), Transcription Factor Binding Sites (TFBS). There are also some works considering that weak motifs also appears in the upstream regions of miRNAs^27^,^28^. Sequence motifs can be detected from software tools, including MEME^29^, DMINDA^30^. Here we try to analyze the motifs around the m^6^A sites, and then employ them as classification features.

Positive and negative sequences were inserted into a general suffix tree. Then all the substrings were listed if it only appeared in the positive sequences or negative ones. We set the least length as 4. So the appearance of these substrings was selected as motif features. If it appeared in one sequence, the feature value was set as 1. Otherwise, the value is 0. Following this process, every sequence was represented as a Boolean vector.

### Ensembles of Support Vector Machine classifiers

Ensemble classifiers were considered to work well on the weak classification problems. However, if the training set was not big enough, ensemble classifiers may cause over-fitting and had weak generalization. Support vector machine (SVM) was always employed for the “small sample size problem”. Structural risk minimization brings good generalization for support vector machine. In order to improve the prediction performance and avoid the over-fitting problem, we proposed a novel ensemble support vector machine strategy for m^6^A prediction.

Ensemble classifier consists of several basic classifiers, and outputs the voting results of the basic classifiers. Research works have agreed that diversity of basic classifiers would improve the voting performance. Here we employed three different SVM classifiers and combined them as an ensemble one. Diversity of the three different SVM classifiers ought to be as more as possible, while accuracy of every SVM classifier need be maintained.

The first two classifiers, namely SVM-PseKNC and SVM-motif, were built based on SVM by using PseKNC and motif features as the inputs, respectively. Although these proper sequence features could be helpful for DNA/protein function prediction, it is believed that numerical features would miss sequence information. Finding good features for the DNA/protein sequence is still empirically difficult and a challenge for the weak classification problems. So some researchers proposed string kernel SVM for the DNA/protein sequence classification problems. Optimized gapped kmers were embedded in the kernel computation, and numerical feature extraction was avoided before SVM classification. GkmSVM^15^ is a software tool, which can deal with DNA sequences directly as training samples. Here we employed GkmSVM as the third basic classifier.

**Figure 2:**
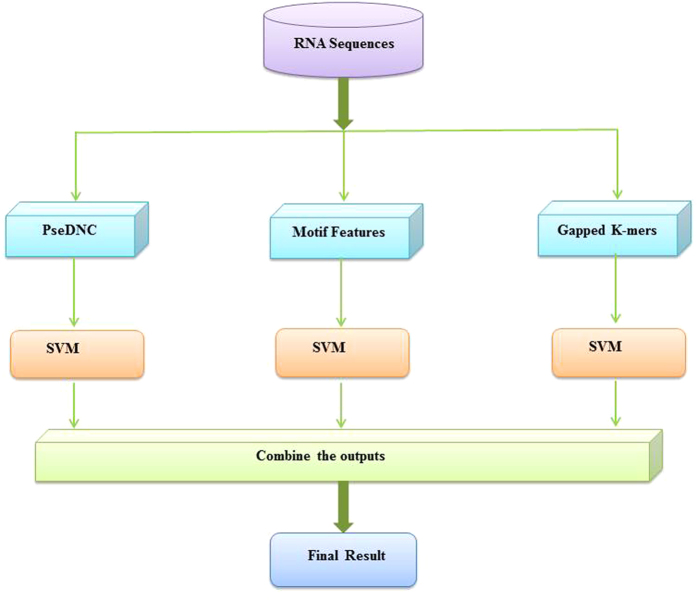
The workflow of RAM-ESVM.

Figure 2 shows the prediction process with the ensemble SVM classifiers. The three basic classifiers votes for the final result. We set different weights to the three basic classifiers as following,


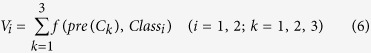


where *V*_*i*_ is the voting score for the RNA sample belonging to the class_*i*_ (m^6^A sites or non- m^6^A sites), *f*(*pre*(*C*_*k*_), Class_*i*_) is the score function defined as


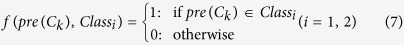


The final prediction is determined by.


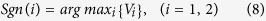


Sgn(*i*) is argument that maximizes the voting score *V*_*i*_.

### Why and when will voting win?

Here we try to analyze the 3 classifiers’ voting strategy. Suppose that the accuracies of the 3 classifiers are *p*_*1*_, *p*_*2*_, *p*_*3*_ (0.5 < {*p*_*1*_, *p*_*2*_, *p*_*3*_} < 1), respectively. So the accuracy of the voted ensemble independent classifiers would be


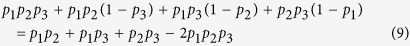


If *p*_*1*_ ≈ *p*_*2*_ ≈ *p*_*3*_ = *q*, the accuracy of ensemble classifier would be 3*q*^2^ − 2*q*^3^. It is easy to prove that 3*q*^2^ − 2*q*^3^ > *q*. Since 0.5 < *q* < 1, *q*(2*q* − 1)(*q* − 1) < 0, it’s obvious 3*q*^2^ − 2*q*^3^ > *q*. Therefore, we can conclude that if the three basic classifiers are independent and approximately equally accuracy, the voting result would be better than the single classifier. In this work, our three basic classifiers employed different features and performed among ~70% accuracy. So the voting strategy could improve the performance.

### Performance evaluation

All the methods were evaluated with sensitivity (*Sn*), specificity (*Sp*), Accuracy (*Acc*) and the Mathew’s correlation coefficient (*MCC*), which are expressed as


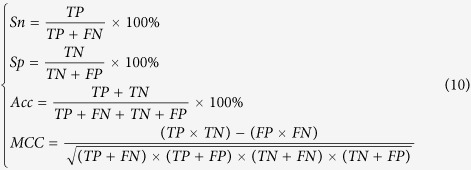


where *TP, TN, FP*, and *FN* represent true positive, true negative, false positive, and false negative, respectively.

## Additional Information

**How to cite this article:** Chen, W. *et al*. Detecting N^6^-methyladenosine sites from RNA transcriptomes using ensemble Support Vector Machines. *Sci. Rep.* **7**, 40242; doi: 10.1038/srep40242 (2017).

**Publisher's note:** Springer Nature remains neutral with regard to jurisdictional claims in published maps and institutional affiliations.

## Figures and Tables

**Table 1:** Comparison of different parameters for identifying m^6^A sites.

Parameters	Sn (%)	Sp (%)	Acc (%)	MCC
32D	64.27	55.78	60.02	0.20
98D	70.00	63.42	66.71	0.33
motif	66.25	78.56	72.41	0.45
PseDNC	71.08	60.21	65.65	0.31

**Table 2:** Comparison of SVM with other classifiers for identifying m^6^A sites.

Classifiers	Parameters	Sn (%)	Sp (%)	Acc (%)	MCC
Naïve Bayes	motif	84.92	50.49	67.71	0.38
	PseDNC	74.98	51.87	63.43	0.27
Random Forest	motif	66.64	75.59	71.11	0.42
	PseDNC	65.72	60.52	63.12	0.26
J48	motif	62.74	68.94	65.84	0.32
	PseDNC	62.89	51.26	57.08	0.14
KNN	motif	32.36	86.91	59.64	0.23
	PseDNC	57.84	54.39	56.12	0.12
SVM	motif	66.25	78.56	72.41	0.45
	PseDNC	71.08	60.21	65.65	0.31

**Table 3:** Performance of ensemble SVM and the single SVMs.

Parameters	Sn (%)	Sp (%)	Acc (%)	MCC
motif	66.25	78.56	72.41	0.45
PseDNC	71.08	60.21	65.65	0.31
gksvm	72.03	77.39	74.71	0.49
Ensemble SVM	78.93	77.78	78.35	0.57

**Table 4:** Comparative results for identifying m^6^A sites between different methods.

Predictor	Sn (%)	Sp (%)	Acc (%)	MCC
M6A-HPCS	77.35	67.41	72.38	0.45
RAM-ESVM	78.93	77.78	78.35	0.57

**Table 5:** The original enthalpy, entropy and free energy values of the dinucleotides.

Dinucleotide	Enthalpy	Entropy	Free energy
GG	−12.2	−29.7	−3.26
GA	−13.3	−35.5	−2.35
GC	−14.2	−34.9	−3.42
GU	−10.2	−26.2	−2.24
AG	−7.6	−19.2	−2.08
AA	−6.6	−18.4	−0.93
AC	−10.2	−26.2	−2.24
AU	−5.7	−15.5	−1.10
CG	−8.0	−19.4	−2.36
CA	−10.5	−27.8	−2.11
CC	−12.2	−29.7	−3.26
CU	−7.6	−19.2	−2.08
UG	−7.6	−19.2	−2.11
UA	−8.1	−22.6	−1.33
UC	−10.2	−26.2	−2.35
UU	−6.6	−18.4	−0.93
